# Molecular typing of human adenoviruses among hospitalized patients with respiratory tract infections in a tertiary Hospital in Guangzhou, China between 2017 and 2019

**DOI:** 10.1186/s12879-021-06412-0

**Published:** 2021-08-03

**Authors:** Xinye Wang, Dawei Wang, Sajid Umar, Sheng Qin, Qiong Ling, Gregory C. Gray, Yuntao Liu

**Affiliations:** 1 Global Health Research Center, Duke Kunshan University, Kunshan, China; 2 Faculty of Medicine, School of Medical Sciences, University of New South Wales, Sydney, NSW Australia; 3 Emergency Department, The Second Affiliated Hospital of Guangzhou University of Chinese Medicine, No. 111 Dade Road, Yuexiu District, Guangzhou, China; 4 Laboratory Department, The Second Affiliated Hospital of Guangzhou University of Chinese Medicine, Guangzhou, China; 5 Division of Infectious Diseases, Duke University, School of Medicine, DUMC Box 102359, Durham, NC 27710 USA; 6 Duke Global Health Institute, Duke University, Durham, NC USA; 7 Program in Emerging Infectious Diseases, Duke-NUS Medical School, Singapore, Singapore

**Keywords:** Adenovirus, PCR, Genotyping molecular epidemiology, Respiratory diseases

## Abstract

**Background:**

Human Adenoviruses (HAdVs) cause a wide array of illnesses in all age groups. They particularly cause frequent morbidity among children. In China, human adenovirus types 3, 4, 7, 11, 14, 21, and 55 have caused at least seven outbreaks since 2000. However, limited studies are available regarding the epidemiological patterns and diversity of HAdVs types among hospitalized patients with respiratory tract infections (RTIs).

**Methods:**

To understand the epidemiology and subtype distribution of HAdV infections associated with RTIs in China, nasal swab (NS) clinical samples were collected from 4129 patients in a Guangzhou hospital between August 2017 and October 2019. PCR, sequencing, and phylogenetic analysis were performed on these specimens to identify HAdV subtypes.

**Results:**

HAdV was successfully sequenced in 99 (2.4%) of the 4129 NS specimens, with the highest HAdV prevalence (6.3%) found in children between the ages of 5 and 10 years. Among HAdV-positive specimens, the most prevalent genotypes identified were HAdV-B3 (55.6%) and HAdV-B7 (25.3%). The most common symptoms in the HAdV-infected patients were fever (100%), cough (80.8%), and rhinorrhea (71.8%). HAdV infections were detected throughout the year with a relatively higher prevalence in summer.

**Conclusion:**

All ages suffer adenovirus infections, but young children are at the greatest risk. This study data demonstrates that at least three species of HAdVs (species B, C, and E) are circulating in Guangzhou City, China. As antiviral therapies and type-specific vaccines become available, such epidemiological data will be useful in guiding therapy and public health interventions.

**Supplementary Information:**

The online version contains supplementary material available at 10.1186/s12879-021-06412-0.

## Background

Human adenoviruses (HAdV) are non-enveloped double-stranded DNA viruses. They belong to the family *Adenoviridae.* Approximately 103 genotypes of HAdV have been documented to date. They are organized into seven species (A-G) [1, 2]. HAdV is a highly contagious pathogen that causes various clinical illnesses, including upper and lower respiratory tract infections, bronchitis, pneumonia, conjunctivitis, and acute gastroenteritis [3]. All age groups of people are susceptible to HAdV infections, while children, immunocompromised patients, cardiovascular disease patients, and military trainees are at a higher risk for developing severe disease [3–5]. It has been reported that HAdV is responsible for at least 5–10% of pediatric and 1–7% of adult respiratory tract infections (RTIs) [6]. HAdV infections have a worldwide distribution, but the distribution of adenovirus subtypes often differs by geographical region or human population. Among all HAdVs, types 3, 4, 7, 14, 21, and 55 often cause severe infections and have been linked to outbreaks globally [7–11]. HAdV-associated outbreaks are more likely to occur in closed and crowded conditions, such as schools, hospitals, or military recruits [7, 9].

In recent years, an increasing number of HAdV outbreaks have been reported in China. An acute respiratory infection (ARI) outbreak caused by a re-emergent isolate of HAdV55 occurred in Shanxi Province in 2006 [12]. An outbreak of febrile respiratory illness caused by HAdV-14p1 occurred in Gansu Province in 2011 [13]. Two outbreaks of acute respiratory diseases caused by HAdV-7 were detected in military training camps, one in Hubei Province and another in Shaanxi Province between 2012 and 2013 [7]. HAdV-B3 was also frequently reported as the common cause of epidemic ARI outbreaks in China [14]. However, limited data are available regarding the epidemiological and clinical features of HAdV in hospitalized patients. In this study, we sought to determine the prevalence and subtypes of HAdVs among hospitalized patients with RTIs in Guangzhou, China. Such epidemiological data are useful to health professionals regarding decisions in employing appropriate therapy and adopting effective prevention strategies for adenovirus control.

## Materials and methods

### Patients and specimens

Nasal swab (NS) specimens (*n* = 4129) were initially obtained from hospitalized patients with RTIs at the Second Affiliated Hospital of Guangzhou University of Chinese Medicine between August 2017 and October 2019. These patients were recruited for participation in the hospital’s study if they met the following inclusion criteria: (i) had a fever (temperature ≥ 37.3 °C, measured at the hospital) with the apparent respiratory symptom(s); (ii) did not have the bacterial infection (patients with white blood cell count > 12 × 10^9/L, or procalcitonin (PCT) > 0.5 ng/L were excluded from this study). Individuals who met the inclusion criteria were consented (parental consent required for adolescents under the age of 18) and invited to complete a brief questionnaire about their demographics and symptoms.

### Subject sample collection

Each participant permitted the collection of one NS specimen (the swab stayed in the nose for 15 s), which was placed in viral transport media (COPAN Diagnostics Inc., Italy), stored in the icebox, and then transported to the hospital’s laboratory. Specimens were preserved at − 80 °C until further processing.

At the hospital’s laboratory, all clinical NS specimens (*n* = 4129) were first screened with the commercially available Immunofluorescence assay (respiratory viral panel 1 screening and identification kit, Light DiagnosticTM, Chemicon International Inc., Temecula, USA) targeting adenovirus, influenza A and B virus, parainfluenza 1–3, and respiratory syncytial virus (RSV). A total of 117 HAdV-positive specimens were detected using the Immunofluorescence assay. No HAdV-positive casses were co-infected with other respiratory viruses (e.g., Influenza A/B virus, PIV1-3, or RSV). Later these HAdV-positive specimens were sent to the Duke Kunshan University (DKU) One Health Research Laboratory to be used for this study.

### Detection of HAdV with real-time PCR

At the DKU One Health Research Laboratory, DNA was extracted from HAdV-positive specimens and eluted in 60 μl of elution buffer using a QIAamp MinElute Virus Spin Kit (Cat. No.57704, Qiagen Inc., Hilden, Germany). Extracted DNA was then tested for adenovirus by real-time quantitative polymerase chain reaction (qPCR) assays using a BioRad SsoAdvanced Universal Probes Supermix (Bio-Rad Laboratories, Richmond, CA) as previously described [15] on a Mic qPCR Cycler (BioMolecular Systems, EI Cajon, CA). Positive and negative template controls were included in each qPCR run. The qPCR cycling was run as follows: 90 °C for 2 min, 95 °C for 3 min, followed by 40 cycles of 95 °C for 15 s, and 60 °C for 30s. Samples with quantification cycles (Cq) values < 38 were considered positive for adenovirus.

Nested PCR targeting the HAdV hexon gene’s hyper-variable regions 1–6 (HVR1–6) was performed for genotyping (predicted amplicon size: 764–896 base pairs (bp) [16]. The outer primers used in the first-round amplification were AdhexF1 (5′-TGTAAAACGACGGCCAGT-TICTTTGACATICGIGGIGTICTIGA-3′) and AdhexR1 (5′-CTGTCIACIGCCTGRTTCCACA-3′). The inner primers used in a second PCR were AdhexF2 (5′-GGYCCYAGYTTYAARCCCTAYTC-3′) and AdhexR2 (5′-GGTTCTGTCICCCAGAGARTCIAGCA-3′). Nested PCR was conducted in a 50 μl volume comprising 5 μl of 10x PCR buffer (−Mg), 0.2 μl (50 μM) of each primer, 1.0 μl of dNTP Mix, 1.5 μl of 50 mM MgCI2, 0.2 μl of Taq DNA Polymerase, 0.5 μl of first nested-PCR product, and 41.4 μl of double-distilled water. Cycling conditions were employed as follows: 94 °C for 2 min, followed by 36 cycles at 94 °C for 1 min, 45 °C for 1 min, and 72 °C for 2 min, and a 5 min extension at 72 °C. PCR products were analyzed on 2% agarose gels and sent for sequencing.

### Sequence and phylogenetic analysis

Hexon gene nucleotide sequences from the specimens collected in this study were aligned with multiple reference strains available in GenBank (accession numbers are listed in Supplementary Table 1) using ClustalW. The phylogenetic tree was constructed by the neighbor-joining method with bootstrap analysis (*n* = 1000) by MEGA7.0 software.

### Statistical analysis

STATA version 15.0 (StataCorp LP, College Station, TX) was used for statistically analysis. The demographic data was analyzed for statistical significance using the Chi-square test or Fisher’s exact test as appropriate. Kruskal-Wallis test was employed for comparisons between two or more groups. The two-tailed value of *P* < 0.05 was considered as statistically significant.

## Results

### Molecular characterization of HAdV

#### qPCR results

Among the 117 immunofluorescence-positive HAdV specimens previously detected by the collaborated laboratory, 104 (88.9%) specimens were detected as HAdV-positive with qPCR at DKU One Health Research Laboratory. The average Cq value was 21.5 (Min: 13.7; Max: 37.4).

#### Sequence and phylogenetic analysis

**Fig. 1:**
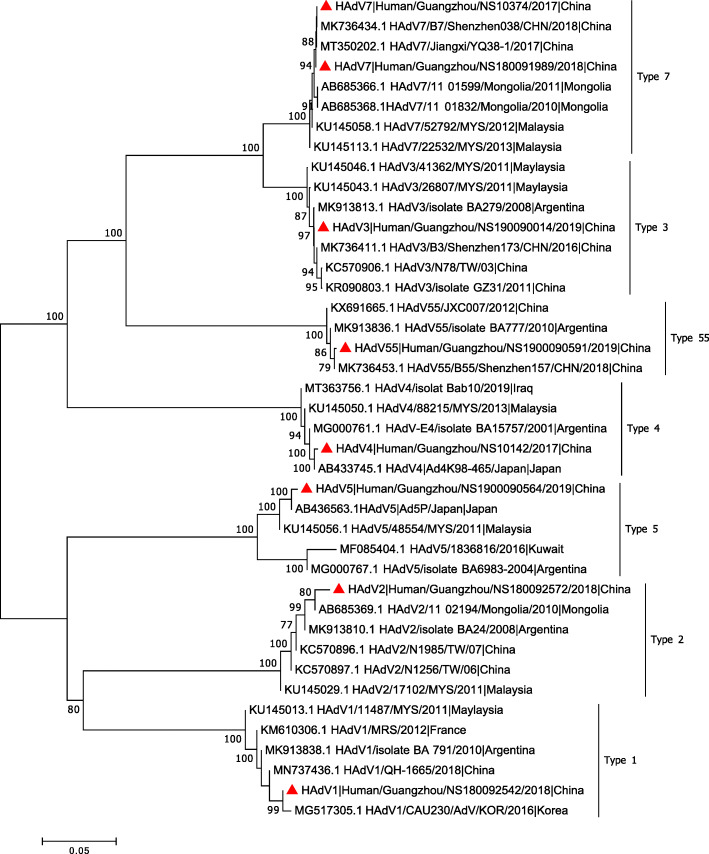
Phylogenetic analysis of the hexon gene of HAdV strains identified in patients hospitalized with respiratory tract infections in Guangzhou between 2017 and 2019. The representative strains detected in this study are marked with red solid triangles. Other reference strains were gathered from previous publications (Supplementary Table 1). The phylogenetic tree was constructed with a neighbor-joining tree method and p-distance model using MEGA version 7 (http://www.megasoftware.net). Bootstrap values were calculated on 1000 replicates, and values < 70% are not shown. The tree is drawn to scale, with branch lengths measured in the number of substitutions per site

To further analyze the HAdV genotype, the hexon gene from 104 HAdV positive specimens was amplified by conventional PCR in this study. Ninety-nine (95.2%) specimens were successfully genotyped. Eight out of 99 sequences selected as representative sequences (see details in supplementary material) which were further aligned with other reference strains (partial Hexon gene) (Fig. 1). The sequences of the hexon genes from patients’ clinical specimens have been deposited in GenBank under the accession numbers MZ220513-MZ220520. The phylogenetic analysis indicated that 84 patients with HAdV belonged to species B, 7 patients with HAdV belonged to species C, and 8 patients with HAdV belonged to species E (Table 3). Within the HAdV-B species, type 3, 7, and 55 were identified. It is worth noting that all the HAdV-B strains identified in this study were highly identical to strains detected in Shenzhen and Jiangxi City, suggesting the genetic conservation of some HAdV-B viruses in different areas of China. Additionally, HAdV-B3 and HAdV-B7 were the most prevalent types in this study. Within the HAdV-C species, types 1, 2 and 5 were identified. All the HAdV-C species generated from this study seem to be more closely related to HAdV-C viruses circulated in neighboring countries. For example, the HAdV-C1 strain in this study was nearly identical to the strain CAU230/AdV/KOR/2016 found in South Korea. The HAdV-C5 strain identified in this study was identical to a Japanese HAdV virus. Study findings indicated that Species B, C, and E (at least seven subtypes) circulated simultaneously in Guangzhou in 2017–2019.

### Demographic data of the hospitalized patients

The 99 HAdV-positive pateints identified in this study included 66 (63.67%) males and 33 (33.33%) females. Their median age was 4 years, range 2 months to 63 years. The HAdV-positive patients were distributed across age groups: Age groups < 5, 5–10, 10–19, and ≥ 19 years, accounted for 3.8, 6.3, 0.6, 0.16% of positives, respectively (Table 1). Distribution of HAdV detection rate among the different age groups was significant different (*p*-value < 0.05). The highest prevalence of HAdV infections was found among children who were between 5 and 10 years (6.3%), followed by those under five years (3.8%). There was no significant difference in HAdV-positivity observed between males and females.

**Table 1:** Demographic characteristics of HAdV-positive (successfully typed) hospitalized patients in Guangzhou, China between August 2017 and October 2019. Patients were enrolled from the Second Affiliated Hospital of Guangzhou University of Chinese Medicine

Characteristics	Number of Patients*	Number of patients positive for HAdVs	Percentage of patients positive for HAdV	P-value
Age (years)^a^				< 0.05
< 5 years	1485	57	3.80%	
5 - < 10 years	600	38	6.30%	
10- < 19 years	169	1	0.60%	
≥ 19 years	1855	3	0.20%	
Gender				0.107
Male	2416	66	2.70%	
Female	1693	33	2.00%	

Note: * Of the 4129 patients, 20 patients had information missing on age, gender, and clinical information, and were thus excluded from the analysis. ^a^ Kruskal-Wallis Test was used for comparison among age groups

### Clinical feature of HAdV-infected patients

Among 99 HAdV-positive patients, two patients lacked clinically relevant information except fever and were not described in Table 2. Clinical diagnosis included pneumonia (38.1%), acute bronchitis (24.7%), acute upper respiratory tract infections (AURTI) (18.6%), and gastroenteritis (4.1%). It is worth noting that approximately 79% of pneumonia patients were found among children under ten years in age. The most prevalent clinical signs were fever (100%), cough (82.5%), rhinorrhea (69.1%), and expectoration (62.9%), while the other clinical presentation were sore throat (19.6%) and diarrhea (8.2%). No HAdV-associated deaths were reported.

**Table 2:** Clinical characteristics of patients who had adenovirus (HAdVs)-positive (successfully typed) nasal swab specimens

Characteristics	HAdV positive (%) (***N*** = 97)
**Diagnosis**	
Pneumonia	37 (38.1)
Acute bronchitis	24 (24.7)
Acute tonsillitis	3 (3.2)
AURTI	17 (17.5)
Gastroenteritis	4 (4.1)
**Symptoms**	
Fever	97 (100.0)
Cough	80 (82.5)
Rhinorrhea	67 (69.1)
Expectoration	61 (62.9)
Sore throat	19 (19.6)
Diarrhea	8 (8.2)
**Prognosis**	
Totally recovery	97 (100)
Death	0 (0)

Note: Among 99 patients with HAdV positive, two patients lacked clinical symptom information except fever and were not included in the above table

### Clinical characteristics of HAdV genotypes

HAdV genotypes differed in their characteristics (Table 3). In addition to fever symptom described in inclusion criteria, the most common clinical manifestations of all HAdV genotypes were cough (50–100%), and rhinorrhea (50–100%). The comparison among the seven HAdV types revealed that HAdV-B7 caused more severe diarrhea than other HAdV types. The majority (94.6%) of pneumonia patients were found to be associated with HAdV-B species (including types 3, 7, and 55). Unexpectedly, three out of four gastroenteritis patients were found in patients who were infected with HAdV-B7.

**Table 3:** Clinical and laboratory characteristics of patients who had a human adenovirus (HAdVs)-positive nasal swab specimen, by HAdV type

Characteristics	HAdV B3* (***n*** = 53)	HAdV B7 (***n*** = 25)	HAdV B55 (***n*** = 4)	HAdV E4 (***n*** = 8)	HAdV C1 (***n*** = 2)	HAdV C2 (***n*** = 3)	HAdV C5 (***n*** = 2)
***Symptoms***							
Fever	53 (100.0)	24 (100.0)	4 (100.0)	8 (100.0)	2 (100.0)	3 (100.0)	2 (100.0)
Cough	45 (84.9.4)	21 (84.0)	4 (100.0)	4 (50.0)	2 (100.0)	2 (66.7)	2 (100.0)
Rhinorrhea	38 (71.7)	15 (60.0)	4 (100.0)	6 (75.0)	1 (50.0)	2 (66.7)	2 (100.0)
Sore throat	9 (17.0)	5 (20.0)	1 (25.0)	3 (37.5)	1 (50.0)	0 (0.0)	0 (0.0)
Expectoration	36 (67.9)	13 (52.0)	3 (75.0)	3 (37.5)	2 (100.0)	2 (66.7)	2 (100.0)
Diarrhea	2 (3.8)	5 (20.0)	0 (0.0)	1 (12.5)	0 (0.0)	0 (0.0)	0 (0.0)
***Diagnosis***							
URTI	10 (18.9)	5 (20.0)	1 (25.0)	0 (0.0)	0 (0.0)	0 (0.0)	2 (100.0)
Acute bronchitis	12 (22.6)	7 (28.0)	1 (25.0)	2 (25.0)	1 (50.0)	1 (33.3)	0 (0.0)
Pneumonia	21 (39.6)	12 (48.0)	2 (50.0)	1 (12.5)	1 (50.0)	0 (0.0)	0 (0.0)
Gastroenteritis	0 (0.0)	3 (12.0)	0 (0.0)	1 (12.5)	0 (0.0)	0 (0.0)	0 (0.0)

Notes: 1) The total number of HAdV-B3 positive specimens is 55, but two patients with information miss on most clinical features were not described. But one thing to note that these two patients were included in this study because they had a fever over 37.3 °C and met other inclusion criteria. 2) URTI (upper respiratory tract infection); HAdV (human adenovirus)

### Seasonal distribution of the HAdV infections

**Fig. 2:**
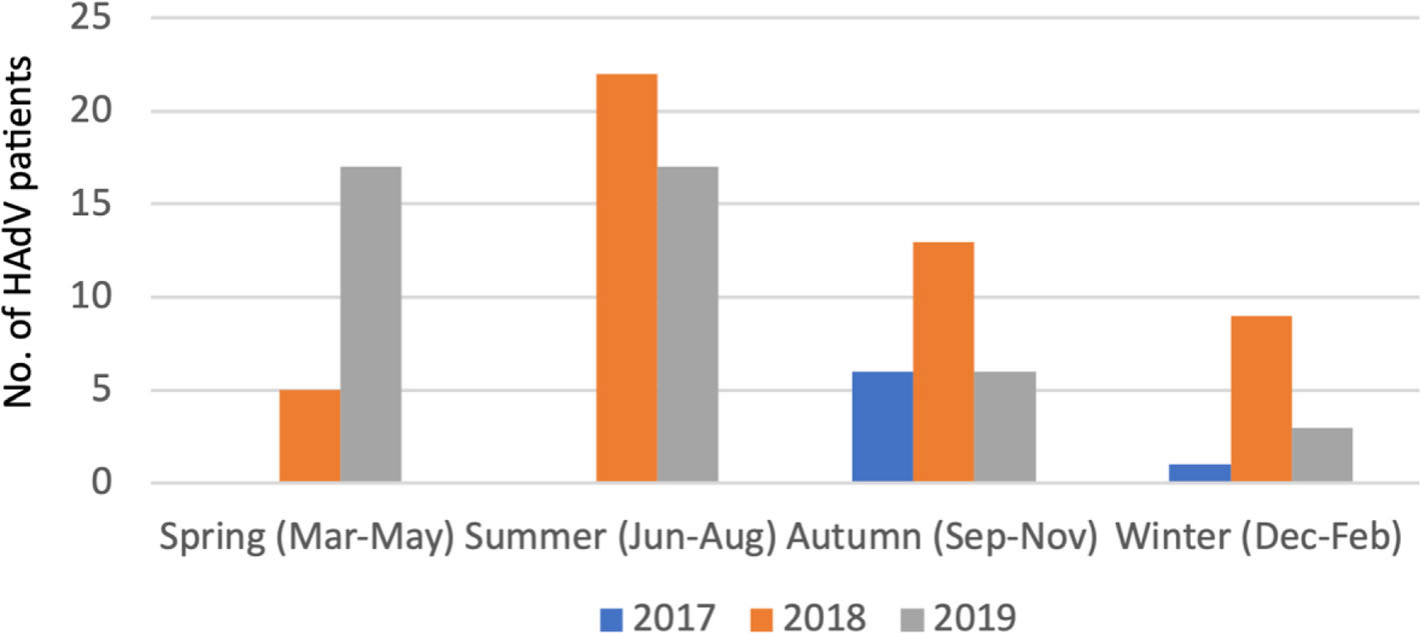
Number of patients with evidence of human adenovirus infection by season

In China, 12 months were classified into four seasons, including Spring (March to May), Summer (June to August), Autumn (September to November), and Winter (December to February). The number of HAdV-positive patients from August 2017 to October 2019 detected in different seasons are depicted in Fig. 2. HAdV infections were detected during each season. However, the highest numbers of HAdV positive patients were found in the summer in both 2018 and 2019 with 22 and 17 HAdV patients, respectively.

## Discussion

The present study recorded the epidemiological distribution of circulating HAdV strains among hospitalized patients with RTIs between 2017 and 2019 in Guangzhou City, China.

In this study, the overall positive rate of HAdV was 2.4%, which is consistent with the positive rate (2.0–6.1%) found in hospitalized patients with acute viral respiratory infection in recent reports in China, Israel, and Switzerland [3, 17–20]. However, two previous studies conducted in the Northern part of China showed a higher HAdV prevalence (10.4–20.1%) than our study [21, 22]. These findings indicate that HAdV prevalence may differ by geographic locations. Such differences in HAdV prevalence could be influenced by a number of other factors, including clinical sample type, patients’ ages, patients’ occupations, patients’ underlying diseases, and sampling period during the patients’ illnesses.

HAdVs are considered one of the major causes of acute respiratory diseases in children and adults worldwide [6, 23, 24]. HAdVs are more prevalent in young children and responsible for approximately 2–10% of pediatric RTIs annually [20, 22]. Our findings are consistent with such worldwide observations in that we found 96% (95 of 99) of the study’s HAdV-positive patients to be less than ten years of age.

Our study revealed that although HAdV infections were detected throughout the year, the prevalence peaked in the summer. This is consistent with findings of Chen et al. [25] during 2012–2013. But it was not consistent with that of studies conducted in Northern China [21] and Mexico [26]. In Tanzania and Switzerland, HAdV infections were observed during all seasons of the year with no clear seasonality demonstrated [19, 27]. These difference in HAdV prevalence between seasons are interesting and bear future study as seasonal risk could influence future employment of HAdV vaccines which are in development in China [28].

In this study, seven HAdV types were identified: HAdV-B3, HAdV-B7, HAdV-B55, HAdV-C1, HAdV-C2, HAdV-C5, and HAdV-E4. Among these types, HAdV-B3 and HAdV-B7 were most prevalent which is consistent with other reports in Asia [29–31]. The majority (94.6%) of HAdV types detected in this study were of species B. HAdV-B (e.g., 3, 7, and 55) has been continuously reported to be associated with more severe acute respiratory disease than other HAdVs species [2, 14, 32]. Additionally, HAdV-B3 and -B7 were the subtypes that have been frequently found in Shenzhen and Guangzhou in recent years [3, 25, 29, 33]. These two cities are 139.3 km adjacent to each other and are both located in the Guangdong province of China. HAdV-B3 and B7 identified in this study were also highly identical to strains detected in Shenzhen, which indicates the conservation of hexon genes of these two subtypes within Guangdong province. Only seven patients were found to have HAdV-C, and eight patients were found to have HAdV-E infection during the study period.

Previously, three types of HAdV-C (e.g., 1, 2, and 5) were identified in China, although studies of HAdV-C species have been limited [34]. HAdV-C species viruses were identified as the primary pathogens responsible for respiratory tract infections among hospitalized children, particularly among infants under two years of age [34]. Our study is consistent with this in that more than half of HAdV-C-positive patients were observed in children less than two years old. Previous research suggests that recombination events are commonly observed among HAdV-C types [34]. As recombinant HAdV strains have caused epidemics, it seems prudent to monitor for changes in HAdV-C types in China.

Although our study provides crucial molecular evidence regarding the epidemiology and clinical features of HAdV infections in Guangzhou, China, it has several limitations. First, we did not study outpatients and they could have had a different distribution of HAdV subtypes. Second, study samples were first identified using a commercial assay which is not thought to be as sensitive as the qPCR we employed. Hence, we likely only captured data on the specimens with higher viral titers. Thus, the true prevalence of HAdV among hospitalized patients may have been higher and the distribution of HAdV subtypes different. Third, according to the laboratory results provided by the collaborated hospital, we did not find any adenovirus-positive patients in this study infected with influenza A/B, PIV 1–3, and RSV at the same time. In addition, we were unable to rule out all other possible respiratory viruses in specimens except HAdV, influenza A/B virus, PIV1–3, and RSV. Hence, we cannot compare the epidemiological results of single infection and coinfections. Future studies may consider using non-targeted metagenomic sequencing approach to explore more information regarding the coinfection issue.

Adenovirus surveillance is important to China. A recent multicenter, prospective registry study found that HAdV was the third-leading cause of viral infection among community-acquired pneumonia patients in China after influenza viruses and respiratory syncytial virus [35]. Additionally, antivirals [36, 37] and vaccines [28, 38] against adenovirus infections are seen on the horizon for China. Even so, China has not yet established a national surveillance system for HAdVs, which seems to be greatly needed. Nationwide, periodic HAdV surveillance could alert Chinese public health officials of the emergence of pre-pandemic or particularly virulent strains and help them mitigate the threat.

## Supplementary Information

**Additional file 1.** Supplementary Material for Sequence and Phylogenetic Analysis. **Supplementary Table 1.** List of reference human adenovirus (HAdV) strains used in the manuscript’s hexon gene phylogenetic comparisons.

## Data Availability

The datasets used and/or analyzed in the current study are available from the corresponding author upon reasonable request. Accession numbers of sequences used in this study have been listed in supplementary document.
