# Supplementary material for: Molecular typing of human adenoviruses among hospitalized patients with respiratory tract infections in a tertiary Hospital in Guangzhou, China between 2017 and 2019
Source: BMC Infect Dis. 2021 Aug 3;21:748. doi: 10.1186/s12879-021-06412-0 (PMC8330471; doi:10.1186/s12879-021-06412-0)
Supplement: Supplementary file 1 — Additional file 1. Supplementary Material for Sequence and Phylogenetic Analysis. Supplementary Table 1. List of reference human adenovirus (HAdV) strains used in the manuscript’s hexon gene phylogenetic comparisons. [file 12879_2021_6412_MOESM1_ESM.docx]

Supplementary Material

**Molecular Typing of Human Adenoviruses among Hospitalized Patients with Respiratory Tract Infections in a Tertiary Hospital in Guangzhou, China between 2017 and 2019**

Xinye Wang^1, 2^, Dawei Wang^3^, Sajid Umar^1^, Sheng Qin^7^, Qiong Ling^7^, Gregory C. Gray*^1,4,5,6^, and Yuntao Liu*^3^

**Affiliations:**

^1^Global Health Research Center, Duke Kunshan University, Kunshan, China

^2^School of Medical Sciences, Faculty of Medicine, University of New South Wales, Sydney, NSW, Australia

^3^Emergency Department, The Second Affiliated Hospital of Guangzhou University of Chinese Medicine, Guangzhou, China

^4^Division of Infectious Diseases, Duke University, School of Medicine, Durham, North Carolina, USA

^5^Duke Global Health Institute, Duke University, Durham, North Carolina, USA

^6^Program in Emerging Infectious Diseases, Duke-NUS Medical School, Singapore

^7^Laboratory Department, The Second Affiliated Hospital of Guangzhou University of Chinese Medicine, Guangzhou, China

## Sequence and Phylogenetic Analysis

A total of 99 Hexon gene nucleotide sequences from clinical samples were obtained in the study. The BLASTn program (National Center for Biotechnology Information, Bethesda, MD, USA) was used to identify the homologous nucleotide sequences in the GenBank database. We identified seven types of HAdV in our sequences. Additionally, we found that some sequences were nearly identical to each other. We thus selected eight representative sequences from each of the seven HAdV subtypes for phylogenetic analysis.

**Supplementary Table 1.** List of reference human adenovirus (HAdV) strains used in the manuscript’s hexon gene phylogenetic comparisons.

| **GenBank ID** | **Organism** | **Country of origin** | **Collection date** | **Strain Name** |
| --- | --- | --- | --- | --- |
| KU145046.1 | Human adenovirus B3 | Malaysia | 2011-Apr | HAdV3/41362/MYS/2011 |
| KU145043.1 | Human adenovirus B3 | Malaysia | 2011-Mar | HAdV3/26807/MYS/2011 |
| MK913813.1 | Human adenovirus B3 | Argentina | 2008-Oct | BA_279-2008 |
| MK736411.1 | Human adenovirus B3 | China | 2016-Oct | B3/Shenzhen173/CHN/2016 |
| KC570906.1 | Human adenovirus B3 | China | 2003-Jan | N78/TW/03 |
| KR090803.1 | Human adenovirus B3 | China | 2011-Aug | GZ_31_2011 |
| AB685366.1 | Human adenovirus B7 | Mongolia | 2011 | 11_01599/Mongolia/hexon |
| AB685368.1 | Human adenovirus B7 | Mongolia | 2010 | 11_01832/Mongolia/hexon |
| MK736434.1 | Human adenovirus B7 | China | 2018-Jul | B7/Shenzhen038/CHN/2018 |
| MT350202.1 | Human adenovirus B7 | China | 2017-May | Adv7/Jiangxi/YQ38-1/2017 |
| KU145058.1 | Human adenovirus B7 | Malaysia | 2012-Nov | HAdV7/52792/MYS/2012 |
| KU145113.1 | Human adenovirus B7 | Malaysia | 2013-Jul | HAdV7/22532/MYS/2013 |
| MK736453.1 | Human adenovirus B55 | China | 2018-Sep | B55/Shenzhen157/CHN/2018 |
| KX691665.1 | Human adenovirus B55 | China | 2012 | JXC007/2012 |
| MK913836.1 | Human adenovirus B55 | Argentina | 2010-Jul | BA_777-2010 |
| MT363756.1 | Human adenovirus E4 | Iraq | 2019 | Bab10/2019 |
| KU145050.1 | Human adenovirus E4 | Malaysia | 2013-Jan | HAdV4/88215/MYS/2013 |
| MG000761.1 | Human adenovirus E4 | Argentina | 2001-Sep | BA_15757-2001 |
| AB433745.1 | Human adenovirus E4 | Japan | - | Ad4K98-465/Japan |
| AB436563.1 | Human adenovirus C5 | Japan | - | Ad5p/Japan |
| KU145056.1 | Human adenovirus C5 | Malaysia | 2011-Apr | HAdV5/48554/MYS/2011 |
| MF085404.1 | Human adenovirus C5 | Kuwait | 2016-Nov | 1836816/2016/Kuwait |
| MG000767.1 | Human adenovirus C5 | Argentina | 2004-Apr | A_6983-2004 |
| AB685369.1 | Human adenovirus C2 | Mongolia | 2011 | 11_02194/Mongolia/hexon |
| MK913810.1 | Human adenovirus C2 | Argentina | 2008-Jun | BA_24-2008 |
| KC570896.1 | Human adenovirus C2 | China | 2007-May | N1985/TW/07 |
| KC570897.1 | Human adenovirus C2 | China | 2006-Apr | N1256/TW/06 |
| KU145029.1 | Human adenovirus C2 | Malaysia | 2011-Feb | HAdV2/17102/MYS/2011 |
| KU145013.1 | Human adenovirus C1 | Malaysia | 2011-Feb | HAdV1/11487/MYS/2011 |
| KM610306.1 | Human adenovirus C1 | France | 2012 | MRS-2012 |
| MK913838.1 | Human adenovirus C1 | Argentina | 2010-Jul | BA_791-2010 |
| MN737436.1 | Human adenovirus C1 | China | 2018-Nov | QH-1665/2018 |
| MG517305.1 | Human adenovirus C1 | South Korea | 2016 | CAU230/AdV/KOR/2016 |
